# Intestinal volvulus in utero causing torsion of dilated bowel with ileal atresia: a case report

**DOI:** 10.1186/s40792-023-01645-4

**Published:** 2023-04-25

**Authors:** Chiyoshi Toyama, Yuki Segawa, Shigeo Iijima, Takeshi Murakoshi, Keigo Nara

**Affiliations:** 1 Department of Pediatric Surgery, Hamamatsu University School of Medicine, University Hospital, 1-20-1, Handayama, Hamamatsu, Shizuoka 431-3192 Japan; 2 Department of Pediatrics, Hamamatsu University School of Medicine, University Hospital, 1-20-1, Handayama, Hamamatsu, Shizuoka 431-3192 Japan; 3 Department of Obstetrics and Gynecology, Seirei Hamamatsu General Hospital, Sumiyoshi, 2-12-12, Naka Ward, Hamamatsu, Shizuoka 430-8558 Japan

**Keywords:** Intestinal atresia, In utero, Intestinal volvulus, Dilated bowel, Torsion

## Abstract

**Background:**

In utero intestinal volvulus with intestinal atresia is a rare and life-threatening condition that can cause torsion of the dilated bowel. The management and outcomes of this disease remain unclear.

**Case presentation:**

A 19-year-old woman noticed a decrease in fetal motion at 35 weeks. Fetal ultrasound showed dilated fetal bowel and the whirlpool sign. The patient was referred to our hospital for an emergency cesarean section. The neonate’s abdomen was dark and severely distended, and a laparotomy was performed. Necrotic ileum and cord-type intestinal atresia (Type II) were observed in the dilated terminal ileum. The necrotic ileum was resected, and a second-look surgery was performed the following day. Then, we anastomosed the remaining intestine, and the total intestine length was 52 cm. There were no surgical complications, and the patient was discharged without requiring total parenteral nutrition or fluid infusion. The patient’s height and weight were within the − 2 standard deviation range of the growth curve at 5 months.

**Conclusions:**

Emergency and appropriate management of intestinal volvulus in utero causing torsion of the dilated bowel resulted in good outcomes in a patient with intestinal atresia. Perinatal physicians should be aware of this emergency condition and plan their treatment approach accordingly.

## Background

In utero intestinal volvulus is a life-threatening condition, and delayed diagnosis contributes to a high incidence of morbidity and mortality [1, 2]. Dilated bowel torsion in intestinal atresia is a recognized postnatal emergency complication [3], which necessitates various therapeutic procedures depending on the condition of the intestines. However, in utero cases are extremely rare, and the management and outcomes remain unclear.

Prenatal diagnosis of intestinal atresia is frequently characterized by bowel distension and can be accompanied by meconium peritonitis [4]. However, the complications associated with in utero intestinal atresia are poorly documented, and the need for prompt intervention is not widely acknowledged.

Herein, we present the case of a successfully treated patient with ileal atresia and intestinal volvulus in utero with subsequent torsion of the dilated bowel.

## Case presentation

**Fig. 1:**
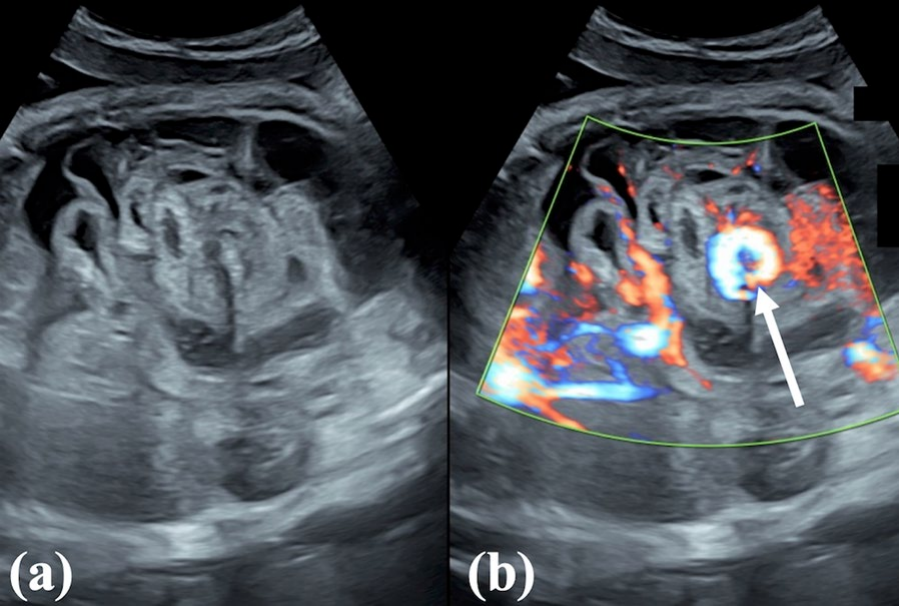
Fetal ultrasound: **a** fetal bowel is dilated with some ascites. **b** Vessels of the bowel are twisted (whirlpool sign, arrow)

**Fig. 2:**
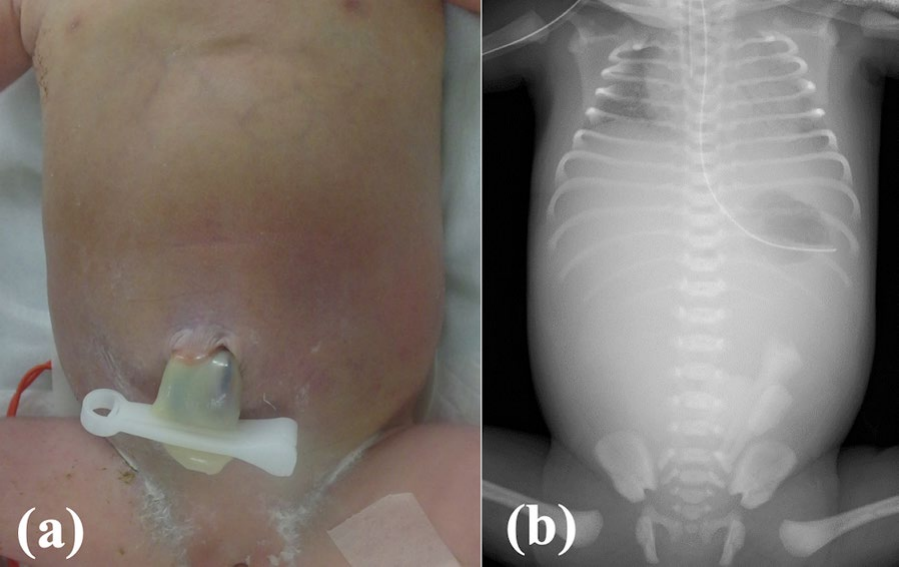
General appearance and the X-ray at birth: **a** newborn’s abdomen was dark and severely distended. **b** X-ray shows gasless abdomen

The patient was a 19-year-old pregnant woman without any prior abnormalities during her pregnancy. A decrease in fetal motion was noticed at 35 weeks. Fetal ultrasound showed dilated fetal bowel and the whirlpool sign (Fig. 1). The patient was referred to our hospital the same day. Cardiotocography showed a decreased variability and absence of deceleration. Because of fetal indications, such as bowel ischemia, an emergency cesarean section was performed. A girl was delivered at 35 weeks with a birth weight of 2752 g. The Apgar scores were 3 and 7 at 1 min and 5 min, respectively. The neonate’s abdomen was dark and severely distended (Fig. 2a). The neonate was intubated immediately. The abdominal X-ray was gasless with a distended abdomen (Fig. 2b). In the blood test, the white blood cell count was 37,520/μL, and the hemoglobin level was 9.6 g/dL. The patient’s aspartate transferase and alanine transferase levels were elevated at 185 U/L and 41 U/L, respectively. There were no findings of renal dysfunction or coagulopathy.

**Fig. 3:**
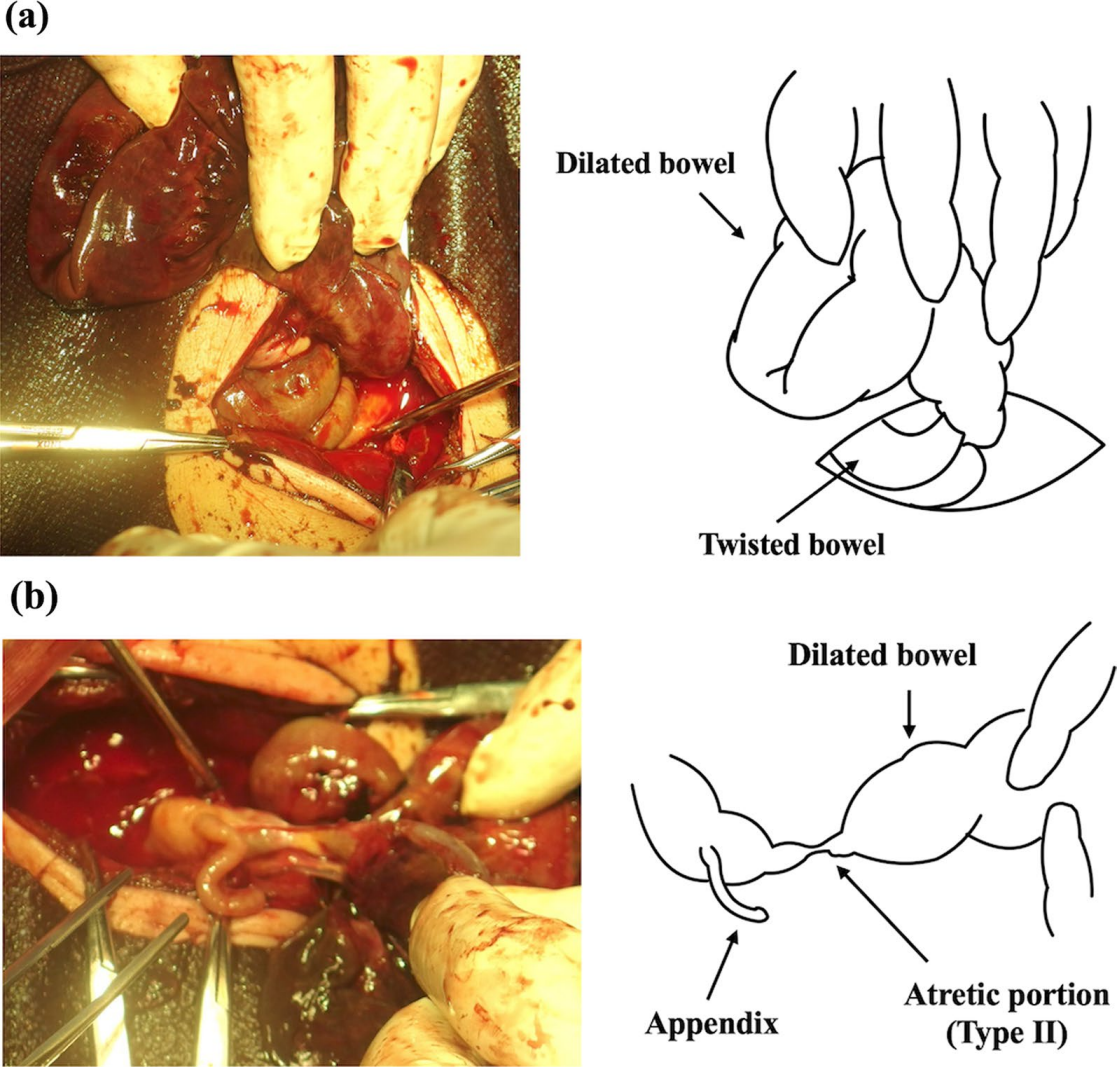
Surgical findings: **a** dilated ileum is twisted and necrotic. **b** Cord-type ileal atresia is demonstrated at the terminal ileum

**Fig. 4:**
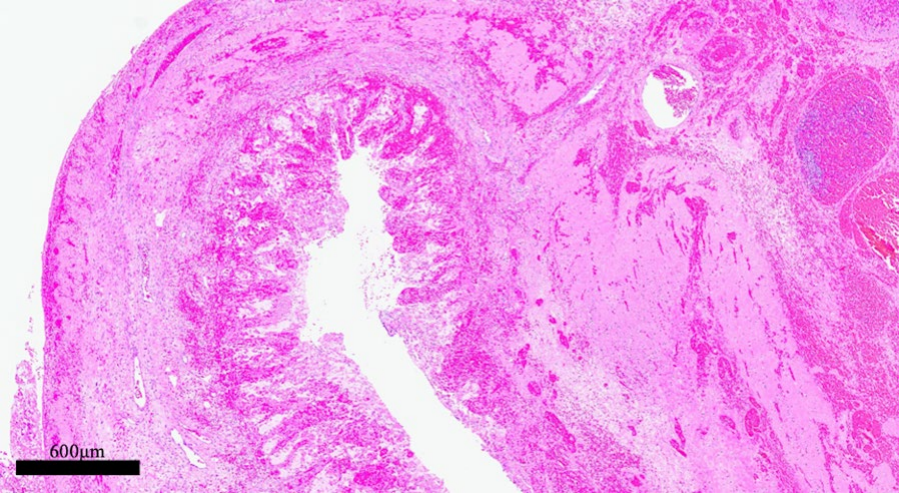
Pathological findings of intestine in first operation: the hemorrhage within mucosa and inflammation in all layers are noted

We performed a laparotomy on the newborn 2 h after birth (5 h after the mother arrived at our hospital). We observed massive ascites with dark fluid, and almost the entire small intestine was twisted around the axis of the dilated intestine. (Fig. 3a). While de-twisting the ileum, a cord-type intestinal atresia (Type II) was detected in the dilated terminal ileum (Fig. 3b). We resected only the obvious necrotic ileum (40 cm) while excluding uncertain ischemic regions. Bilateral intestinal stamps were temporally closed and returned to the abdominal cavity. Then, 24 h after the initial surgery, a second-look surgery was performed, and we resected a 5-cm necrotic proximal intestine and a cord-type atretic portion on the distal intestine. Consequently, the remaining small intestine was 52 cm. Nearly the entire jejunum and approximately 2 cm of the terminal ileum were preserved after resection. We anastomosed these segments end-to-end to avoid an ileostomy and to preserve the ileocecal valve and intestine. The pathological findings of resected intestines were hemorrhage within mucosa and inflammation in all layers. No other abnormalities were noted (Fig. 4). The neonate’s respiratory function worsened due to elevated bilateral diaphragms, and the abdominal wall was treated using the silo procedure with an open abdomen. Respiratory function improved 1 week after the second surgery, and the abdominal wall was closed.

The patient started feeding at 2 weeks after the anastomosis. There were no surgical complications, and the patient was discharged 3 months later without requiring total parenteral nutrition or fluid infusion. The patient showed growth beyond the growth curve, and at 5 months of age, her height and weight were within the − 2 standard deviation range.

## Discussion

Since intestinal volvulus in utero with torsion of the dilated bowel in patients with ileal atresia is rare, surgical treatments and their outcomes are uncertain. Our patient was treated with emergency interventions, which preserved the growth and development of intestinal autonomy.

Intestinal volvulus in utero is reported as a rare neonatal surgical emergency [1, 5–7]. Prenatal diagnosis and sonographic findings include dilated bowel loops, the whirlpool, coffee bean, kinked loop signs, and polyhydramnios [2, 8]. The sensitivity and specificity of the whirlpool sign after birth are 45.4% and 99%, respectively [9]. Similarly, the specificity in utero may be high. In a literature review by Ohuoba et al., it was reported that of the fourteen in utero intestinal volvulus cases, nine were born by vaginal delivery and five by cesarean section, with all cases surviving [1]. However, the relationship between the length of the remaining intestinal tract and the delivery method is unclear. In the present case, despite the absence of severe distress during fetal monitoring, we elected to perform an emergency cesarean section due to fetal indication. Almost the entire small intestine was twisted around the axis of the dilated intestine. Our choice of delivery method might explain the good outcome for our patient.

Midgut malrotation is the most common cause of intestinal volvulus in utero [5]. We found four cases of intestinal volvulus with intestinal atresia [1, 10–12]. Reported cases include intestinal atresia with volvulus, and meconium peritonitis and intestinal atresia with volvulus and intussusception [13]. The whirlpool sign was detected in two cases (50%). Unlike volvulus with malrotation, the whirlpool sign might be difficult to detect because the degree and mechanisms of torsion vary. In our case, we made a diagnosis with sufficient ultrasonographic evidence, which enabled us to treat the patient promptly.

Dilated bowel torsion in intestinal atresia after birth necessitates emergency surgery [3, 14], requires various technically challenging surgical techniques, and long-term outcomes should be considered. Thus, to prevent short bowel syndrome, it is essential to resect necrotic areas or malfunctioning bowel, and also plicate the intestine and evaluate the need for an ileostomy. The same principle applies in utero, and we believe emergency procedures should be mandatory protocol. We performed immediate laparotomy while being careful not to resect uncertain intestinal segments. Although the bowel was not entirely healthy, we performed intestinal anastomosis during the second-look surgery. It is difficult to preserve the ileocecal valve if an ileostomy is created. We believe immediate and appropriate interventions, such as intestinal autonomy without needing home parenteral nutrition, are crucial for a good prognosis.

A prenatal diagnosis of intestinal atresia, characterized by bowel distension, is common, and most cases are delivered electively. However, an emergency cesarean section might be required if an intestinal volvulus is present. Obstetricians, neonatologists, anesthesiologists, surgeons, and other physicians involved in perinatal care should be aware of this disease and be prepared for emergency intervention.

## Conclusions

The patient in the current case presented with intestinal atresia, which caused intestinal volvulus in utero with torsion of the dilated bowel. However, emergency interventions and appropriate surgical procedures resulted in good outcomes. Perinatal physicians should aim to understand intestinal atresia in utero and plan accordingly in an emergency.

## Data Availability

Not applicable.
